# Dual-Method Characterization and Optimization of Drilling Parameters for Picosecond Laser Drilling Quality in CFRP

**DOI:** 10.3390/polym16182603

**Published:** 2024-09-14

**Authors:** Zhao Zheng, Yao Ma, Zhonghe Wang, Siqi Liu, Chunting Wu

**Affiliations:** Jilin Key Laboratory of Solid-State Laser Technology and Application, Changchun University of Science and Technology, Changchun 130022, China; zz944380512@163.com (Z.Z.); w1125740757@126.com (Z.W.); lsq2574609610@163.com (S.L.)

**Keywords:** dual-method characterization, picosecond laser, drilling quality, carbon fiber-reinforced polymer, response surface methodology

## Abstract

Carbon fiber-reinforced polymer (CFRP), known for its light weight, high strength, and corrosion-resistant properties, is extensively used in the lightweight design of satellite components, the optimization of electronic device casings, and the processing of high-performance composite materials in the defense sector. This study employs picosecond laser drilling technology for the precision machining of CFRP, demonstrating its advantages over traditional mechanical drilling and other unconventional methods in significantly reducing the heat-affected zone (HAZ) and enhancing hole wall quality. The optimization of laser power, scanning speed, and fill times via response surface methodology (RSM) significantly reduced the hole wall taper to 4.160° and confined the HAZ to within 18.577 μm, thereby enhancing machining precision. The actual test results show that the deviations in the hole taper and HAZ width were 5.0% and 2.2%, respectively, further verifying the effectiveness of the optimization method. This technique not only improves processing quality but also offers significant industrial application value in the machining of materials for related high-tech fields.

## 1. Introduction

As the demand for lightweight structural products continues to grow, composite materials are attracting increasing attention. Carbon fiber-reinforced polymer (CFRP) is a high-performance composite material that has proven to be a flexible and adaptable engineering material suitable for various applications. This is due to its excellent mechanical and physical properties, including high specific strength, high specific stiffness, excellent thermal stability, and outstanding corrosion resistance [1]. Due to these advantages, CFRPs have become a promising alternative to traditional metal materials in a wide range of industrial fields, including aerospace, automotive, and defense, where structural materials with unique properties are required [2,3]. In the aerospace sector, CFRP is extensively used in critical satellite structures such as load-bearing cylinders, antennas, and camera barrels, replacing metals to achieve lightweight and high-strength designs. The typical thickness range of CFRP used in satellites is from 0.4 mm to 2 mm, with skins or sheets within a thickness range of 0.8 mm becoming a primary focus in processing and application [4].

Although CFRP is typically manufactured into near-net shapes through various molding processes, subtractive manufacturing such as cutting and drilling remains an inevitable part of most product fabrication. As devices such as portable medical equipment, drones, and miniature precision instruments evolve towards miniaturization and integration, light weight and convenience have become primary goals. The smaller component sizes of these compact products necessitate higher precision in small hole machining to meet connection and assembly requirements, driving an increasing demand for high-quality small hole processing. Research into small hole machining processes for CFRP helps improve hole quality, thereby extending the service life of CFRP structures, supporting the development of miniature integrated devices, and broadening the application scope of CFRP materials from structural to mechanical components, necessitating high precision and low damage processing. Bolted connections and riveting are two typical assembly methods that affect the quality of composite material hole machining. Mechanical drilling has become the most crucial operation for processing fiber composites to meet connection requirements. However, traditional mechanical drilling is prone to defects such as delamination, burrs, and cracks at the edges of holes, while also causing significant tool wear and increasing processing costs [5]. Reports indicate that during aircraft assembly, conventional machining-induced delamination causes up to 60% of carbon fiber composite components to be scrapped [6]. Among non-traditional machining methods, technologies such as electrical discharge machining (EDM) [7], abrasive water jet machining (AWJM) [8], ultrasonic machining (USM) [9], and laser processing [10] have shown greater potential.

However, non-traditional machining methods such as electrical discharge machining (EDM) and ultrasonic machining have lower efficiency and produce more hole defects [11]. The resonance frequency matching problem in ultrasonic machining also needs to be addressed [12]. Abrasive water jet machining (AWJM) causes minimal thermal damage, but it is difficult to control processing precision, resulting in high surface roughness and material delamination [13]. Laser machining, as an emerging processing technology, offers advantages such as small scale, high controllability, environmentally friendly processes, no material type restrictions, non-contact processing, and no heat transfer. It is expected to become a viable means of overcoming the precision machining bottleneck of fiber-reinforced composites and could effectively reduce hole defects in CFRP drilling [11]. Laser machining of CFRP involves thermal ablation removal of material by absorbing laser energy. Due to the significant difference in thermal properties between carbon fibers and the matrix material, thermal damage, particularly in the heat-affected zone, is inevitable, significantly reducing the mechanical properties of the material [14,15].

In recent years, researchers have investigated the effects of laser parameters on the heat-affected zone (HAZ). Wolynski et al. [16] conducted perforation studies on CFRP using picosecond lasers at wavelengths of 355 nm, 532 nm, and 1064 nm. They found that under identical process parameters, the quality of perforations made with the 355 nm ultraviolet light was significantly better than those made with green (532 nm) and infrared (1064 nm) lasers. Anzai et al. [17] used infrared and ultraviolet lasers for laser cutting of CFRP and concluded that the cutting quality with ultraviolet lasers was superior to that with infrared lasers. Li et al. [18] also found that ultraviolet laser processing yielded a smaller HAZ.

Apart from laser wavelength, CFRP cutting typically employs two types of lasers—pulsed lasers and continuous-wave (CW) lasers. Previous studies have found that short-pulse or ultra-short-pulse laser sources release high peak power in a short time, rapidly heating the material and directly causing evaporation or plasma generation. This reduces the amount of heat absorbed by the matrix, thereby limiting the expansion of the heat-affected zone (HAZ). Bluemel et al. [19], through a comparison of continuous lasers and pulsed lasers (nanosecond and picosecond), found that short-pulse lasers produced the smallest heat-affected zones and the highest tensile strength. Yongdu Li et al. [20] used a UV femtosecond laser to cut carbon fibers with a thickness of 2 mm, obtaining a HAZ of about 25 µm. They also used a single-factor method to analyze the influence of laser average power, repetition frequency, and scanning speed on the cutting quality. Meiling Chen et al. [21] found that cutting 1 mm-thick CFRP with an 800 nm femtosecond laser can easily achieve a heat-affected zone (HAZ) of less than 10 μm, which is significantly smaller than the HAZ produced by most other laser processing methods for CFRP. Although femtosecond lasers minimize the heat-affected zone (HAZ) due to their ultra-short-pulse duration, picosecond lasers still offer adequate control over thermal effects while providing a faster material removal rate. This balance is crucial for applications where both precision and processing speed are essential. Freitag et al. [22] used a 1.1 kW infrared picosecond laser to cut 2 mm-thick CFRP, achieving thermal damage of less than 20 μm and an effective cutting speed of 0.9 m/min. Salama et al. [23] used a 400 W infrared picosecond laser to drill 6 mm-thick CFRP, achieving a heat-affected zone of less than 25 μm by optimizing process parameters. J. Finger et al. [24] demonstrated that by repeatedly ablating a 350 μm-wide groove, they achieved precise cutting of 2 mm-thick CFRP samples, with a heat-affected zone of less than 5 μm. This indicates that high-power picosecond lasers can play an important role in the efficient and precise machining of CFRP.

Leone et al. found that in the cutting of CFRP sheets, an increase in scanning speed significantly reduces the HAZ and hole taper. Higher scanning speeds reduce thermal accumulation, decreasing thermal damage and improving hole quality [25]. Salama et al. demonstrated that while a higher laser power of 400 W in picosecond lasers aids in effective material removal during drilling 6 mm-thick CFRP, it significantly increases HAZ and hole taper if not coordinated with other parameters such as scanning speed and frequency. Lowering laser power appropriately helps control HAZ and reduces thermal damage [23]. Ouyang et al., through their research on the application of picosecond lasers in CFRP drilling, found that adjusting scanning speed and laser power can effectively control HAZ and hole taper. Increasing scanning speed helps reduce thermal accumulation, thus decreasing the width of the HAZ and the hole taper [26]. Wang et al. studied the effect of fill times on CFRP drilling, discovering that a judicious selection of fewer scanning passes can avoid thermal accumulation, thereby achieving high-quality drilling results [27]. Li et al.’s experiments indicate that appropriately increasing the number of scanning passes helps remove material layer by layer, reducing the expansion of the heat-affected zone and hole taper. However, excessive passes can lead to thermal accumulation, and thus a balance is necessary [28]. Yongdu Li employed a single-factor analysis method to study the effects of average laser power, repetition frequency, and scanning speed on cutting quality, providing a basis for parameter selection [20].

Manufacturers are particularly interested in production efficiency, quality, and cost minimization. These goals often require “optimal” parameter settings. However, the optimal parameter settings for one quality characteristic may deteriorate other quality characteristics. The quality of laser-processed materials is affected by various process parameters, such as laser power, scanning speed, filling times, and the type and thickness of the workpiece [29]. Output measurement characteristics include the heat-affected zone (HAZ), hole taper, and material removal rate [30]. By appropriately selecting and optimizing laser parameters, material quality performance can be improved. A large number of experimental methods are required to study the effects of process parameters on hole morphology and performance, which consumes a significant amount of time, manpower, and resources [31].

Mathematical modeling and the optimization of process parameters in laser processing can improve quality and efficiency while reducing costs. Therefore, researchers have modeled and optimized process parameters in laser processing. The response surface method (RSM) and its variants are the most widely used methods in laser processing modeling [32,33,34,35]. RSM requires specific experimental designs, such as the Taguchi method, central composite design (CCD), or Box–Behnken design (BBD). BBD can minimize the number of experiments and evaluate the quadratic interactions between factors [36]. The Taguchi design allows for smaller experimental groups. In CCD, the estimation of individual effects, quadratic effects, and interaction effects of factors is more accurate because it better understands endpoints and perimeters, generating better quadratic models [37].

The current research mainly focuses on the effects of process parameters on hole morphology and the heat-affected zone, but further studies are needed on the quantitative relationship and precise control of process parameters with hole morphology and the heat-affected zone. This study uses a picosecond laser to create 1.5 mm-diameter holes in 0.8 mm-thick CFRP. The effects of laser power, scanning speed, and filling times on hole quality are investigated using the control variable method. Quality response indicators include the entrance heat-affected zone and hole taper, and the results are quantified. The response surface method is used to design experiments, analyze the main factors affecting hole quality, and experimentally validate the findings. The optimal laser drilling process parameters are determined, providing a theoretical reference for the precise control of CFRP laser processing morphology.

## 2. Experiment

### 2.1. Materials

**Figure 1:**
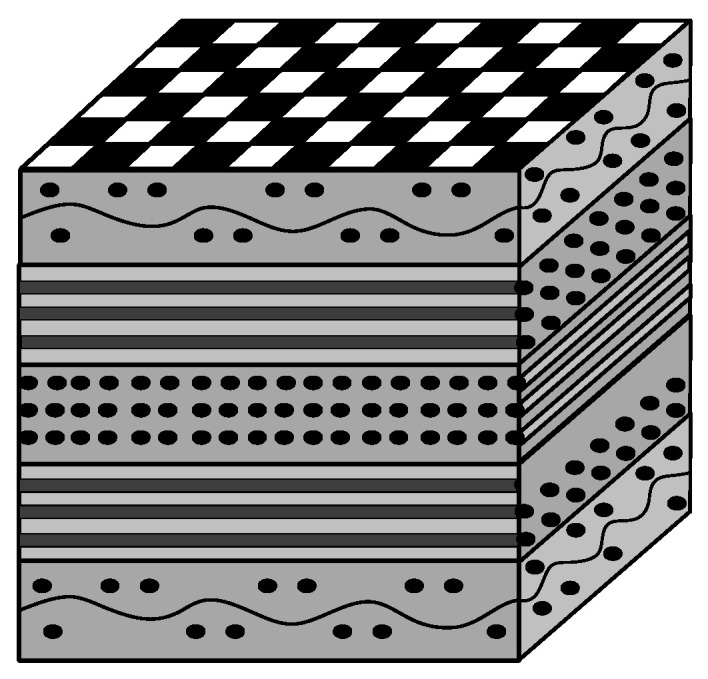
Structure of carbon fiber-reinforced polymer (CFRP).

For this experiment, 0.8 mm-thick CFRP sheet material, manufactured by Toray Composite Materials America, Inc., located in Tacoma, WA, USA, was used as the substrate for the drilling tests. The samples were made from Toray® T300 continuous carbon fibers and epoxy resin, mixed at a volume ratio of 0.6:0.4, and formed using compression molding techniques. The carbon fibers used were the high-strength T300-3k model. The composite was manufactured using high-strength T300-3k carbon fibers and an epoxy resin matrix, employing compression molding techniques. As shown in Figure 1, the CFRP laminate structure has a thickness of 0.8 mm, consisting of five layers. The top and bottom layers are cross-woven, with a thickness of about 0.175 mm, and the internal layers are arranged with 0° and 90° unidirectional tapes, each with a thickness of about 0.15 mm, with a fiber volume fraction of 60%. The significant differences in physical properties between the carbon fibers and the resin matrix in CFRP result in strong anisotropy of the material, making laser processing of CFRP particularly challenging. Its typical thermal properties are shown in Table 1.

### 2.2. Laser Drilling Experimental Setup

**Figure 2:**
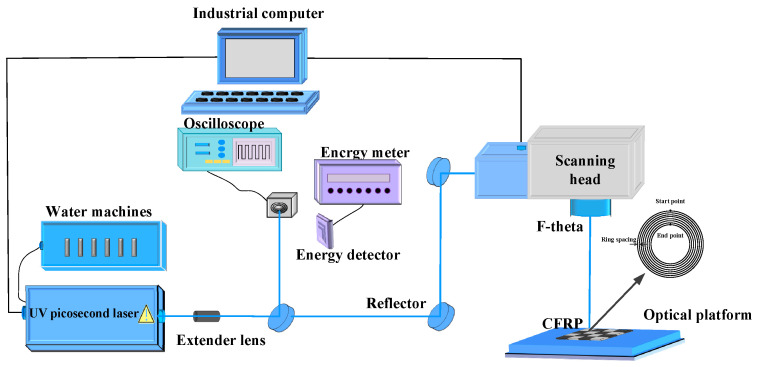
Schematic diagram of laser drilling system experimental setup and scanning method.

The solid-state ultraviolet picosecond laser system used in this experimental study can produce laser pulses with a duration of 10 ps and a wavelength of 355 nm. Other parameters of this laser system are shown in Table 2. This study investigated the effects of laser power, scanning speed, and filling times on the hole taper and the heat-affected zone. The experiment used a coaxial ring drilling method and an outside-to-inside scanning sequence for drilling CFRP, as shown in Figure 2. Under the same experimental conditions, each experimental group underwent three treatments, and the heat-affected zone and entrance and exit diameters of each hole were measured. The average values were calculated, and standard deviations were used as error bars. All experiments were conducted in the air.

## 3. Measurement/Characterization

### 3.1. Hole Taper

**Figure 3:**
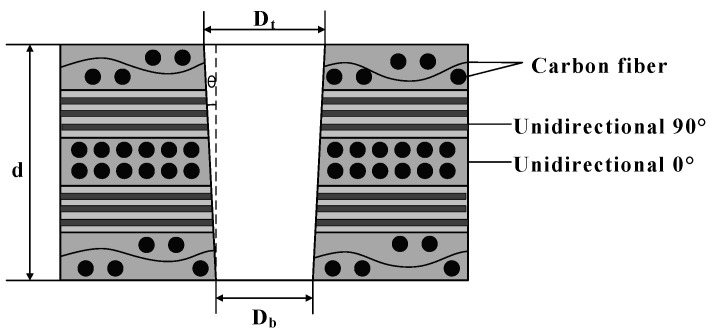
Schematic diagram of hole taper in material cross-section.

Due to the influence of laser beam distribution and sample thickness, there is a certain diameter difference between the entrance and exit diameters of the hole, resulting in a certain taper after laser drilling. As shown in Figure 3, the laser beam ablates material of a certain thickness, and due to the Gaussian beam, a certain taper is produced. As the laser focus decreases along the material thickness (with each decrease being less than the focal depth), a partial shielding zone appears outside the upper surface entrance, and the defocused laser energy has a threshold for removing the shielding zone, thus increasing the entrance diameter. When the energy density does not reach the ablation threshold, the entrance diameter no longer changes. At the same time, the shielding zone at the entrance causes the exit diameter to become smaller, producing a taper [38].

CFRP is an anisotropic material. This means that its physical and mechanical properties vary in different directions. During the laser drilling process, this anisotropy may cause inconsistent ablation rates in different directions, resulting in an elliptical exit hole.

When measuring the exit shape, using a circular selection method will not accurately capture the actual exit area. This approach can lead to selection bias: it may either overextend, covering unnecessary parts, or be too narrow, failing to encompass the entire area. Therefore, in this experiment, we decided to use an elliptical shape to measure and characterize the exit hole size.

**Figure 4:**
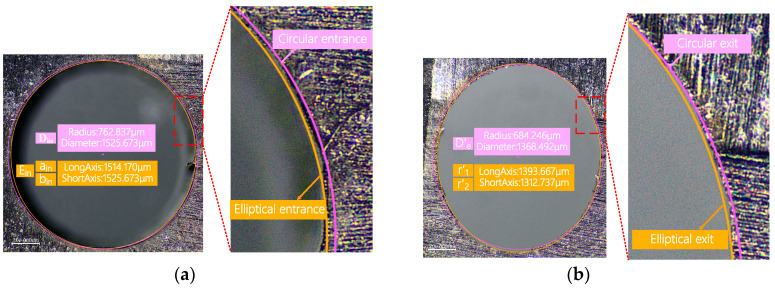
Schematic diagram of quantitative characterization of hole taper. (**a**) Inlet surface of the hole. (**b**) Outlet surface of the hole.

(1) As shown in Figure 4, when observing the optical microscope images around the sample hole, we first measured the entrance diameter of the hole, denoted as Din. For the exit part of the hole, as its shape is closer to an ellipse, we used an elliptical selection frame to determine its diameter. To describe the taper of the hole, we introduced a parameter T. Specifically, T represents the ratio or difference between the exit diameter and the entrance diameter of the hole, characterizing the taper of the hole.

The hole taper T_circle_ is:

(1) $$T_{Circle}=\arctan\frac{D_{\mathrm{in}}-E_{\mathrm{out}}}{2d}=\arctan\frac{D_{\mathrm{in}}-\left(a_{\mathrm{out}}+b_{\mathrm{out}}\right)/2}{2d}$$

Here, D_in_ represents the diameter of the circular hole at the inlet, E_out_ represents the diameter of the elliptical hole at the outlet, and a_out_ and b_out_ represent the major and minor axes of the elliptical hole at the outlet, respectively.

During laser drilling of CFRP, mechanical vibrations and external interferences can cause slight deviations in the laser beam’s path, resulting in the cutting line not following the preset trajectory exactly. Additionally, if the laser beam does not strike the material surface perpendicularly during processing or if there is a deviation during focusing, it can cause uneven energy distribution. This can result in the entrance of the hole being elliptical rather than perfectly circular.

Therefore, in the second method, we use ellipses to simultaneously measure the inlet and outlet of the hole. As shown in Figure 4, T_ellipse_ represents the size of the hole taper. T_ellipse_ is defined as:

(2) $$T_{\mathrm{ellipse}}=\arctan\frac{E_{\mathrm{in}}-E_{\mathrm{out}}}{2d}=\arctan\frac{\left(a_{\mathrm{in}}+b_{\mathrm{in}}\right)/2-\left(a_{\mathrm{out}}+b_{\mathrm{out}}\right)/2}{2d}$$

Here, E_in_ represents the diameter of the elliptical hole at the inlet, E_out_ represents the diameter of the elliptical hole at the outlet, and a_out_ and b_out_ represent the major and minor axes of the elliptical hole at the outlet, respectively.

### 3.2. HAZ

**Figure 5:**
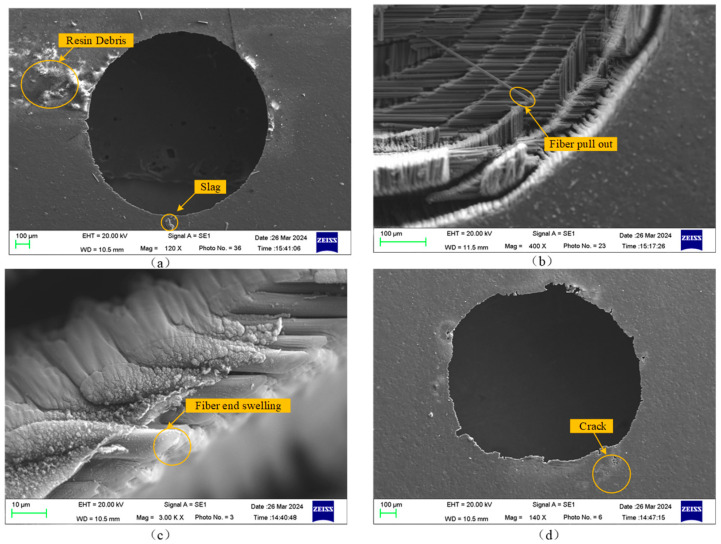
SEM images of typical quality defects in laser drilling of CFRP composites. (**a**) Slag, resin debris. (**b**) Fiber pull-out. (**c**) Fiber end swelling. (**d**) Crack.

Laser processing utilizes a high-energy laser beam to act on materials, functioning as a non-contact machining method. Compared to mechanical machining, it has no mechanical cutting force or tool wear and boasts advantages such as good spatiotemporal controllability, low noise, and no pollution, offering unique benefits in overcoming current CFRP processing issues. Therefore, the laser processing of carbon fiber-reinforced polymer (CFRP) has become a focus of both academic and industrial communities. The laser processing of CFRP mainly relies on the interaction between the laser and the material, which causes the material temperature to rise, removing material through melting or vaporization. However, due to the vastly different thermodynamic properties such as vaporization temperature and thermal conductivity between the resin and carbon fiber composing the CFRP, laser processing of CFRP is prone to defects such as resin debris, slag, fiber pull-out, fiber end expansion, and cracks, as shown in Figure 5.

**Figure 6:**
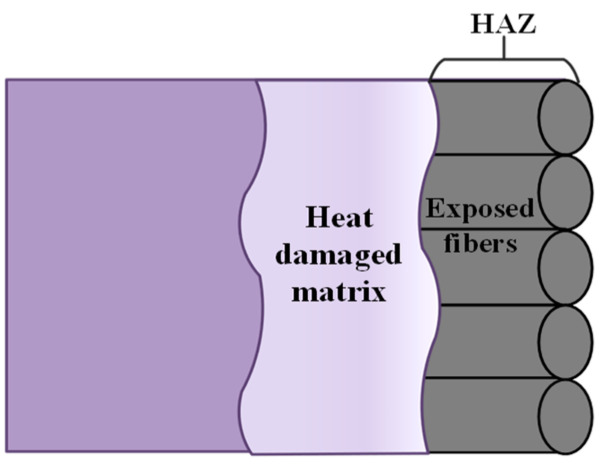
Schematic diagram of HAZ in material cross-section.

Among these, the heat-affected zone (HAZ) is the most significant defect in the laser processing of CFRP. This is due to the vastly different properties of the two materials that compose CFRP. CFRP is composed of carbon fibers and epoxy resins with different components. The thermal properties of the two materials are significantly different, making CFRP an anisotropic material. The evaporation temperature of carbon fibers (3900 K) is much higher than that of epoxy resin (698 K), and carbon fibers have better thermal conductivity [39]. When the laser irradiates the carbon fiber composite material, the resin and carbon fibers absorb energy and vaporize. Part of the energy is rapidly transferred along the carbon fiber axis, causing the internal resin to pyrolyze, forming heat-affected zones such as fiber exposure. Although picosecond lasers have characteristics similar to “cold processing”, processing carbon fiber composites still creates micron-level heat-affected zones (HAZ), which are the most common defects in the processing. As shown in Figure 6 in this experiment, the exposed fiber areas are referred to as the heat-affected zone. When quantifying the heat-affected zone around the hole, the entrance of the hole is used as the quantification object because the surface heat-affected zone contour at the entrance is clearer, making the quantification results more accurate.

The anisotropy of CFRP causes the size and distribution of the heat-affected zone to be highly correlated with the fiber layup direction. Currently, there is no unified standard for representing the heat-affected zone. In this experiment, the heat-affected zone will be quantified using the following two methods.

(1) The first method uses an inscribed ellipse to represent the heat-affected zone, referred to as the Ellipse Method, and its value is denoted as HAZ_ellipse_. This method is based on theoretical analysis and surface morphology. Since the heat-affected zone is approximately elliptical, this method simplifies the representation of the heat-affected zone.

**Figure 7:**
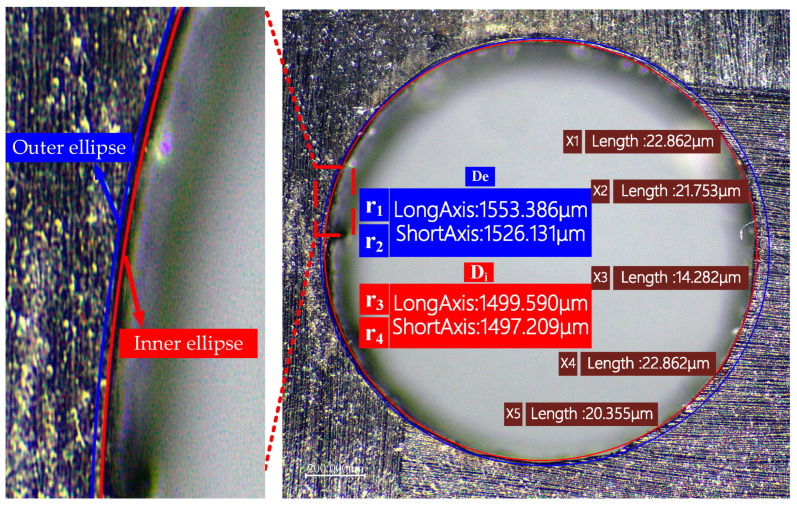
Schematic diagram of quantitative characterization of HAZ around the hole.

As shown in Figure 7, by measuring the major and minor semi-axes of the ellipse from the surface morphology image, the average values of the major and minor axes of the outer ellipse (D_e_) and the inner ellipse (D_i_) are obtained. The heat-affected zone can be calculated by finding the difference between these two averages and then dividing it by 2. For example, using Equation (3), the HAZ_ellipse_ calculated by the Ellipse Method is:

(3) $${\mathrm{HAZ}}_{\mathrm{ellipse}}=\frac{\mathrm{De}-\mathrm{Di}}{2}=\frac{\left(r_{1}+r_{2}\right)-\left(r_{3}+r_{4}\right)}{2}$$

In the equation, r_1_ and r_2_ represent the major and minor axes of the outer ellipse, while r_3_ and r_4_ represent the major and minor axes of the inner ellipse.

(2) As shown in Figure 6, based on the optical microscope images around the sample hole, the HAZ width was measured for five sets of data, and the final HAZ width was the average of these five sets.

(4) $${\mathrm{HAZ}}_{\mathrm{line}}=\frac{\left(X_{1}+X_{2}+X_{3}+X_{4}+X_{5}\right)}{5}$$

## 4. Single-Factor Experimental Results and Analysis of Picosecond Laser Hole Taper

To investigate the impact of single-factor variations on laser-drilled CFRP, this paper employed picosecond laser drilling experiments. The discussion below focuses on the effects of laser power, scanning speed, and fill times under picosecond laser conditions on hole taper and the heat-affected zone. Other laser parameters, including the repetition frequency (500 kHz) and pulse width (10 ps), were held constant.

### 4.1. Effect of Laser Power on CFRP Hole Taper

**Figure 8:**
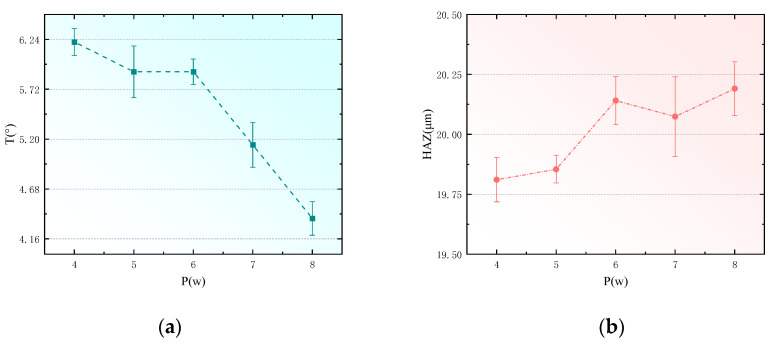
Effect of laser power on hole taper and heat-affected zone. (**a**) Effect of laser power on hole taper. (**b**) Effect of laser power on heat-affected zone.

To analyze the effects of laser power on the taper and heat-affected zones of holes drilled in CFRP, this study utilized a pulse width of 10 ps, pulse frequency of 500 kHz, scanning speed of 500 mm/s, and fill times of 1000 N, with experiments conducted at laser powers of 4 W, 5 W, 6 W, 7 W, and 8 W. Other than the laser power, all selected laser parameters remained consistent. The measured data for the hole taper and the width of the heat-affected zones were fitted, as shown in Figure 8.

From Figure 8a, it is observed that as the power increases (from 4 W to 6 W), the heat-affected zone (HAZ) progressively enlarges. When processing CFRP with picosecond lasers, the expansion of the heat-affected zone (HAZ) is influenced by the thermal accumulation effect, material characteristics, geometric structure, and the heterogeneity between carbon fibers and resin. Due to the high pulse frequency and short pulse intervals of the laser, residual heat accumulates within the material. Particularly when continuous pulses target the same area, the insufficiently cooled heat leads to significant thermal accumulation, thereby enlarging the HAZ. Additionally, the thermal conductivity along the direction of the carbon fibers in CFRP is higher, resulting in uneven heat distribution as laser power increases. Variations in the material’s geometric structure, depth, and absorption characteristics also affect the heat conduction paths. The differing thermal conductivities of carbon fibers and resin further exacerbate the expansion of the HAZ, as the high thermal conductivity of carbon fibers facilitates rapid heat conduction in localized areas, leading to local overheating.

From Figure 8b, it is observed that as the laser power increases from 4 W to 6 W, the hole taper progressively decreases. In the process of picosecond laser hole-making in CFRP, the hole taper can be effectively reduced by optimizing the heat accumulation and material removal efficiency. As the laser power increases, the material removal efficiency increases, and the rapid evaporation and removal reduce the heat diffusion caused by heat conduction, thereby reducing the difference between the upper and lower parts of the hole diameter. High-power lasers can remove hole wall materials more evenly, reduce irregular melting, improve the straightness of the hole wall, and reduce the taper. In addition, as the laser power increases, the thermal effect is more concentrated in the laser action area, reducing the hole diameter change caused by heat diffusion and making the hole shape more regular. In picosecond lasers, the electron-lattice dual temperature model shows that the laser energy is transferred to the lattice through electrons, triggering a violent phase explosion, and the material quickly evaporates into high-energy particles and forms a jet, effectively removing the molten material. At the same time, the laser with Gaussian energy distribution enhances local ablation at the hole entrance, improves the hole wall quality, and further reduces the taper.

### 4.2. Effect of Scanning Speed on CFRP Hole Taper

**Figure 9:**
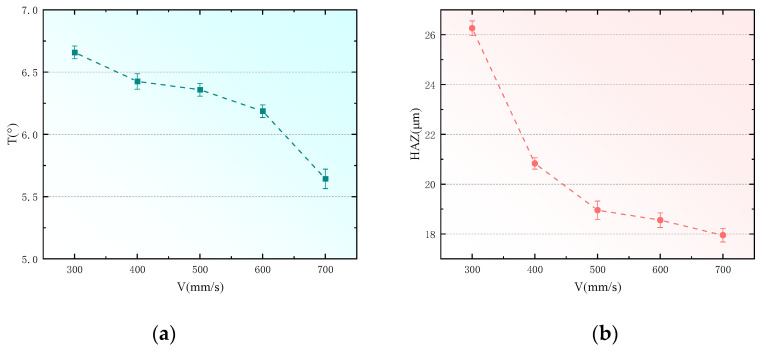
Effect of scanning speed on hole taper and heat-affected zone. (**a**) Effect of scanning speed on hole taper. (**b**) Effect of scanning speed on heat-affected zone.

To analyze the impact of scanning speed on the taper and heat-affected zones of holes drilled in CFRP, this study utilized a pulse width of 10 ps, pulse frequency of 500 kHz, laser power of 6 W, and fill times of 1000 N, with scanning speeds set at 300 mm/s, 400 mm/s, 500 mm/s, 600 mm/s, and 700 mm/s for the experiments. Apart from the scanning speed, all other laser parameters were kept constant. The measured data for the hole taper and the width of the heat-affected zones were fitted, resulting in the outcomes shown in Figure 9.

From Figure 9a, it is evident that during picosecond laser drilling of CFRP, controlling the scanning speed can effectively reduce thermal damage and hole taper. At lower scanning speeds, the laser remains over one area longer, causing the material to absorb excessive heat. This results in a larger heat-affected zone (HAZ), leading to thermal damage such as carbonization, melting, and cracking, and creates uneven material removal, resulting in a larger hole taper. Conversely, increasing scanning speed reduces the time the laser spends on any part of the material surface, decreases energy input, and thus reduces the extent of the heat-affected zone and thermal damage. This allows for more uniform material removal and reduces hole taper. Additionally, at high scanning speeds, heat quickly dissipates, preventing localized overheating and further reducing thermal damage and taper formation. At low scanning speeds, the thermal physical differences between carbon fibers and the resin matrix can lead to excessive carbonization or vaporization of the resin. At high scanning speeds, material removal predominantly occurs through ablation, reducing machining deformation and hole taper.

Figure 9b shows that as the scanning speed increases, the heat-affected zone (HAZ) progressively decreases. In the process of laser processing CFRP, the laser energy density, heat conduction mechanism, and removal method closely affect the size of the heat-affected zone (HAZ). When the scanning speed is low, the laser beam stays in the same area for a longer time, and the material absorbs more energy, resulting in carbonization, melting, and even vaporization. In particular, due to the difference in thermal conductivity between carbon fiber and resin matrix, the resin is more easily decomposed, forming a larger heat-affected zone. Increasing the scanning speed shortens the laser’s residence time per unit area and reduces the local temperature peak, and the heat dissipates before it is conducted to the surrounding area, so the heat diffusion range is reduced. In addition, the transient heat conduction effect causes the heat at high scanning speed to be concentrated on the surface of the material, reducing the heat accumulation effect. In this case, the ablation mechanism dominates the material removal, and the laser energy directly vaporizes the surface material, inhibits the melting process, reduces heat diffusion and thermal damage, and reduces the heat-affected zone.

### 4.3. Effect of Filling Times on CFRP Hole Taper

**Figure 10:**
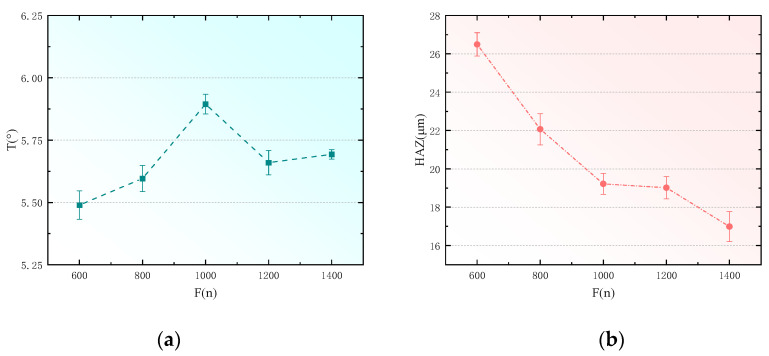
Effect of filling times on hole taper and heat-affected zone. (**a**) Effect of filling times on hole taper. (**b**) Effect of filling times on heat-affected zone.

To analyze the effects of fill times on the taper and heat-affected zones of holes drilled in CFRP, this study utilized a pulse width of 10 ps, pulse frequency of 500 kHz, laser power of 6 W, and scanning speed of 500 mm/s, with fill times set at 600, 800, 1000, 1200, and 1400 for the experiments. Apart from the fill times, all other laser parameters were kept constant. The measured data for the hole taper and the width of the heat-affected zones were fitted, resulting in the outcomes shown in Figure 10.

From Figure 10a, it is observed that in the process of drilling CFRP with picosecond lasers, the hole taper increases progressively with the number of fill times. During the picosecond laser drilling of CFRP, the combined effects of thermal accumulation, material properties, and scanning paths influence the shape and taper of the holes. Each laser fill results in partial energy absorption by the material and conversion into heat; as fill times increase, accumulated heat raises local temperatures, causing material melting and resolidification, altering the shape of the machined area. Carbon fiber conducts heat well, whereas the resin matrix tends to decompose or carbonize at high temperatures. Intensified thermal accumulation leads to carbonization of the resin and damage to the carbon fibers, increasing the taper between the hole bottom and the walls. Additionally, the material removal mechanism changes during the filling process; initial scans vaporize or ablate the surface material to form steep hole walls, but as heat concentrates at the bottom and sides of the hole, molten material resolidifies, reducing removal efficiency and resulting in irregularly tapered holes. Concentric circular scanning paths lead to excessive local heat accumulation due to multiple overlaps, further affecting the material removal efficiency and the shape of the hole, ultimately resulting in a greater taper.

From Figure 10b, it is evident that in the process of picosecond laser drilling of CFRP, optimizing thermal accumulation and dissipation processes, refining material removal, and controlling heat conduction paths effectively reduce the heat-affected zone (HAZ) and enhance machining quality. Employing a strategy of multiple low-energy fills not only reduces energy input during each laser interaction but also increases the scanning intervals, allowing more time for material cooling and thus limiting heat accumulation and depth propagation. This approach reduces localized overheating and carbonization, particularly in resin areas, and by confining heat conduction primarily to the surface layers, it diminishes the risk of deep thermal damage. Additionally, precise surface ablation during multiple fills helps maintain material structural integrity, avoiding thermal stress accumulation and excessive physical damage, thereby improving the quality and precision of the holes.

## 5. Response Surface Design Verification

### 5.1. Response Surface Design

After obtaining the experimental results, a statistical analysis was performed. First, the obtained results were approximated to a response surface-based prediction model. An empirical model based on multiple linear regression was established using response surface methodology (RSM) [40,41]. DOE, ANOVA, and CCD were used to analyze and optimize the input parameters (laser power, scanning speed, and filling times) and their effects on the hole taper and the heat-affected zone. Using this method, an analysis of variance provided a series of response values. This method is widely used for optimizing processing parameters in laser machining.

To establish a relationship between the input factor and output response, response surface methodology was applied. Second-order polynomials, which are non-linear functions having linear, interacting, and power terms, were used in the modeling of several manufacturing processes, as given by Equation (5).

(5) $$y=k_{0}+\overset{n}{\underset{i=1}{\sum }}k_{i}x_{i}+\overset{n}{\underset{i,j=1}{\sum }}k_{i,j}x_{i}x_{j}+\overset{n}{\underset{i=1}{\sum }}k_{i,j}x_{i}^{2}$$

where k_0_ is a free term and coefficients k_i_, k_j_, and k_i,j_ are linear, interacting, and quadratic terms, respectively. xi represents the input parameters, which are laser power, scanning speed, and filling times. y is the output parameters, which are the hole taper and the heat-affected zone. Since input parameters (laser power, scanning speed, and filling times) have different units and different ranges in the experimental data set, in the RSM techniques, the regression equations are not developed on the physical variable. The input process parameters are normalized and known as coded variables, which are dimensionless variables that range from −2 to 2.

Based on the response surface method, a three-factor, five-level experiment was designed using the central composite design (CCD) model in Design Expert 13.0 software. Laser power (P), scanning speed (V), and filling times (F) were taken as the optimization variables, with hole taper and the heat-affected zone as the response variables. A total of 20 experiments were conducted, including 14 axial points and six center points. The coded and actual parameter values for the selected factor levels are shown in Table 3. To avoid systematic error, the experiments were conducted in a random order. The specific experimental parameters and measured results are shown in Table 4.

From the table, it can be seen that the results of the four characterization methods vary similarly with changes in processing parameters, showing good consistency. The hole taper decreases with increasing power, and the heat-affected zone decreases with increasing speed. However, this only indicates that the measurement results of the four methods conform to the processing rules and do not allow for an accurate determination of which method is more precise and reliable from the table. Next, the precision of the four characterization methods will be analyzed in detail using variance analysis.

### 5.2. Optimization of Process Parameters and Experimental Validation

To minimize both the hole taper (T) and the heat-affected zone (HAZ), multi-objective optimization was conducted within the range of the laser drilling parameters studied in this experiment. The optimization range of each parameter was determined through laser drilling experiments. Table 5 shows the selected parameters and their optimization ranges.

To verify the accuracy of the optimization model, experiments were conducted based on the parameter combinations obtained for each evaluation criterion. The feasibility of the optimized laser drilling process parameters was then assessed. The error calculation formula is shown in Equation (6):

(6) $$\Delta H=\frac{\left|H_{0}-H_{1}\right|}{H_{1}}\times 100\%$$

In the formula, H_0_ represents the predicted value of the measurement target, while H_1_ represents the actual value. The relative error of the measurement target is given by these values.

The experimental results and error rates are shown in Table 6. It can be seen that there is a certain error between the experimental results and the predicted results, but the error is within the allowable range (the error may come from machine tool error, model error, measurement error, and material property error). We found that the experimental and predicted values of Plan 3 were consistent, indicating small data errors and a reliable prediction model. Therefore, the subsequent analysis in this study will use the data from Plan 3 as the basis.

**Figure 11:**
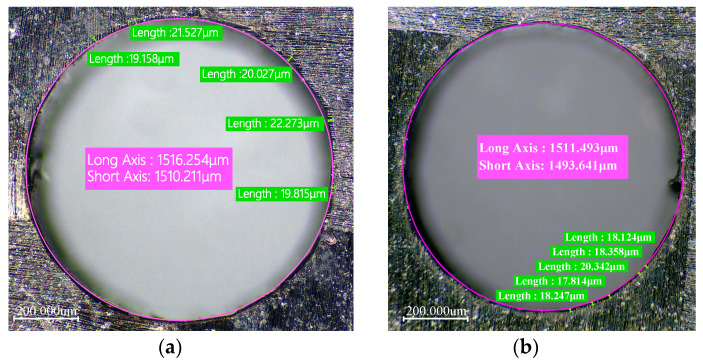
Comparison of inlet heat-affected zone before and after optimization of process parameters. (**a**) Inlet heat-affected zone before optimization. (**b**) Inlet heat-affected zone after optimization.

**Figure 12:**
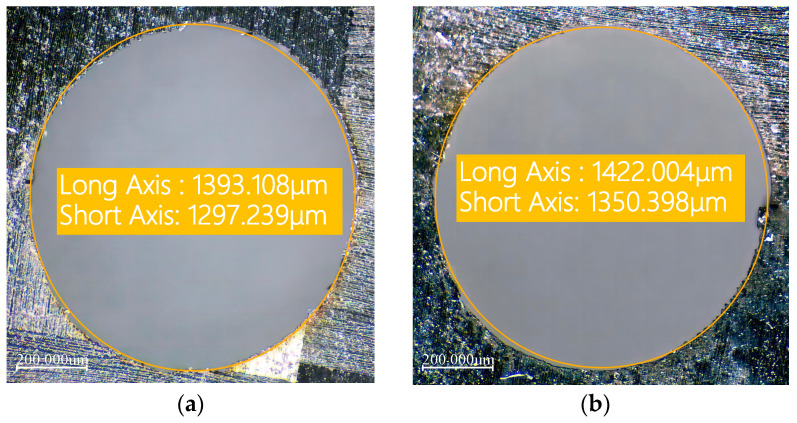
Comparison of hole taper before and after optimization of process parameters. (**a**) Bore taper before optimization. (**b**) Bore taper after optimization.

To verify the optimization effect, experiments were conducted at the 0 level of the response surface experiment (i.e., laser power of 6 W, scanning speed of 500 mm/s, and filling times of 1000 N). Figure 11 shows the comparison of the heat-affected zone width before and after optimization, and Figure 12 shows the comparison of hole taper before and after optimization. It can be seen that the optimization of process parameters is effective.

### 5.3. Mathematical Model and Variance Analysis

#### 5.3.1. Mathematical Model and Variance Analysis of Hole Taper

Based on the experimental data of Plan 3 in Table 4, the hole taper results were calculated. Using response surface analysis, the hole taper and process parameters were fitted into a polynomial to obtain the hole taper process parameter regression prediction model. The p-values of the linear terms (P, F), interaction terms (PV, VF), and quadratic terms (P^2^, V^2^, F^2^) in the model are all less than 0.05, indicating significant terms in the regression equation. The mathematical model for hole taper in Equation (7) is obtained as follows:

T = 5.95756 − 0.410224P + 0.0691275F − 0.284803PV + 0.14743VF − 0.0888774P^2^ + 0.200704V^2^ − 0.0722549F^2^ (7)

As shown in Table 7, the obtained hole taper model was subjected to variance analysis and significance testing. The F-distribution test statistic for the removal rate was 75.90, and the p-value test was used to determine the reliability of the model, with *p* < 0.05 generally being considered significant [42]. In this experiment, the *p*-value for hole taper was <0.0001, indicating that the established model in this study is significantly reliable. The signal-to-noise ratio of the removal rate model was 36.6883 > 4, generally indicating that the model is reliable [43]. The fit degree R^2^ = 0.9856, and the closer this value is to 1, the better the model fit. The predicted fit coefficient Pred.R^2^ = 0.9193 matches well with the adjusted fit coefficient Adj.R^2^ = 0.9726. The model’s lack-of-fit value was not significant, at 0.3218. All indicators suggest that the mathematical model for hole taper fits well [44].

Variance analysis shows that the laser power has an extremely significant impact on the taper of the hole. Its F-value is as high as 306.31 and the *p*-value is less than 0.0001, which directly determines the energy absorbed by the material, which in turn significantly affects the taper of the hole. In comparison, the impact of scanning speed and number of fillings is relatively small; although scanning speed affects the residence time of the laser on the material, its impact on the taper is weak at a high laser power. The number of fillings mainly affects the accuracy and edge quality of the hole but has a limited impact on the overall taper of the hole.

**Figure 13:**
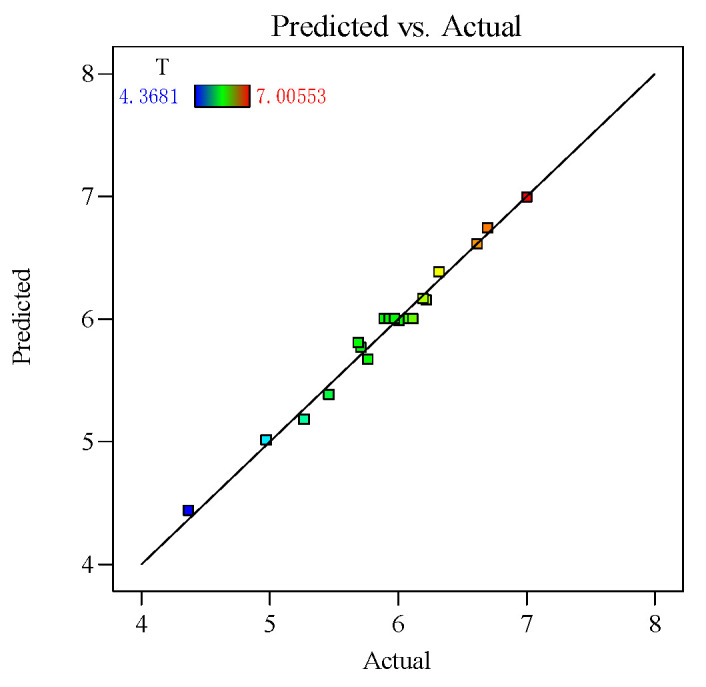
Plot of the actual and predicted values of the hole taper.

To further investigate the reliability of the hole taper process parameter regression model, the predicted values of hole taper were calculated using the fitted hole taper function, and the calculated results were compared with the actual values, as shown in Figure 13. The analysis of the comparison results shows that the data points are distributed around the fitted line, indicating a high degree of fit for the hole taper process parameter regression model, thus proving the model’s accuracy. In summary, the regression model is significant, has a high degree of fit, and has reliable predictive ability.

#### 5.3.2. Mathematical Model and Variance Analysis of the Heat-Affected Zone

Using the same method as constructing the heat-affected zone process parameter regression model, a heat-affected zone process parameter regression model was established. Based on the experimental results, the HAZ regression model was obtained through a fitting analysis of 20 sets of data. The p-values of the linear terms (V, F), interaction terms (PV, PF, VF), and quadratic terms (V^2^, F^2^) in the model are all less than 0.05, indicating significant terms in the regression equation. The mathematical expression is shown in Equation (8):

HAZ = 19.2065 − 2.13321V − 0.7841F + 0.356525PV + 0.4039PF + 0.726225VF + 0.701141V^2^ + 1.07062F^2^ (8)

To verify the effect of process parameters on the dilution rate of the cladding layer, significance testing of the constructed HAZ process parameter regression model was performed using variance analysis. The test results are shown in Table 8.

The analysis of Table 8 yields the following results: The F-value in the variance analysis is 111.35, and the *p*-value is <0.0001, indicating that the regression model is highly significant. The lack-of-fit *p*-value is 0.1381 > 0.05, which is not significant, indicating that the regression model is reliable. The signal-to-noise ratio of the HAZ model is 34.1836 > 4, and the fit degree R^2^ = 0.9901. The closer this value is to 1, the better the model fit. The predicted fit coefficient Pred.R^2^ = 0.9397 matches well with the adjusted fit coefficient Adj.R^2^ = 0.9812. This proves that the regression prediction model is reasonable and can be used for data simulation.

An analysis of variance shows that scanning speed has a very significant impact on the HAZ, with an F-value as high as 584.26 and a p-value less than 0.0001, which indicates that scanning speed is a key factor in controlling the HAZ range. When the scanning speed is low, the laser stays in the same area for a long time, resulting in a larger HAZ; conversely, when the scanning speed is fast, the heat is dispersed, thus reducing the range of the HAZ. In contrast, although laser power affects the energy input, its impact on HAZ is relatively weakened under fast scanning. The F-value is only 1.19 and the p-value is 0.3006, showing a small and insignificant effect. The number of fillings mainly affects the machining accuracy and hole quality, and is less directly decisive for the HAZ range, although multiple fillings may increase the HAZ.

**Figure 14:**
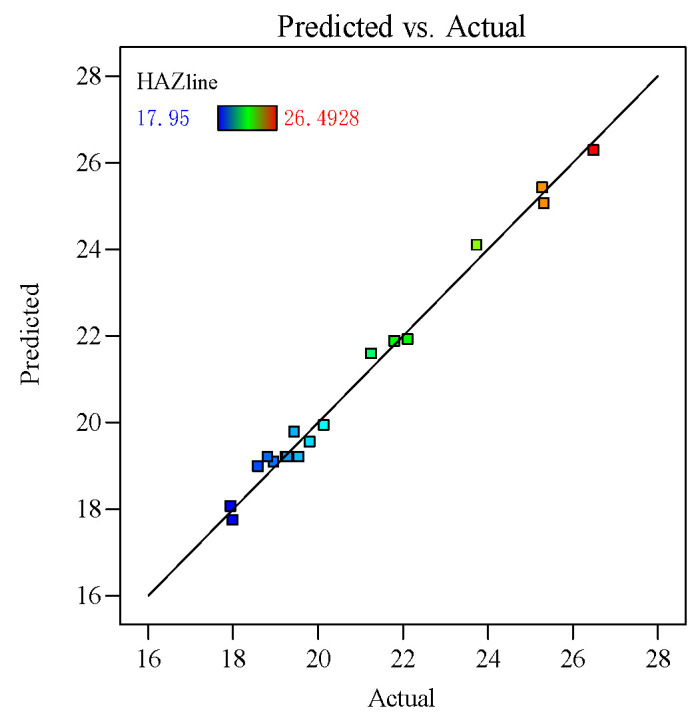
Plot of the actual and predicted values of the heat-affected zone.

To verify the reliability of the constructed HAZ process parameter regression model, Figure 14 shows the actual values versus the predicted values for the HAZ regression model, from which it can be seen that the data points are evenly distributed around the fitted line with no outliers, indicating a high degree of fit and reliable predictive ability.

### 5.4. Effect of Process Parameters on Hole Quality

#### 5.4.1. Effect of Process Parameters on Hole Taper

**Figure 15:**
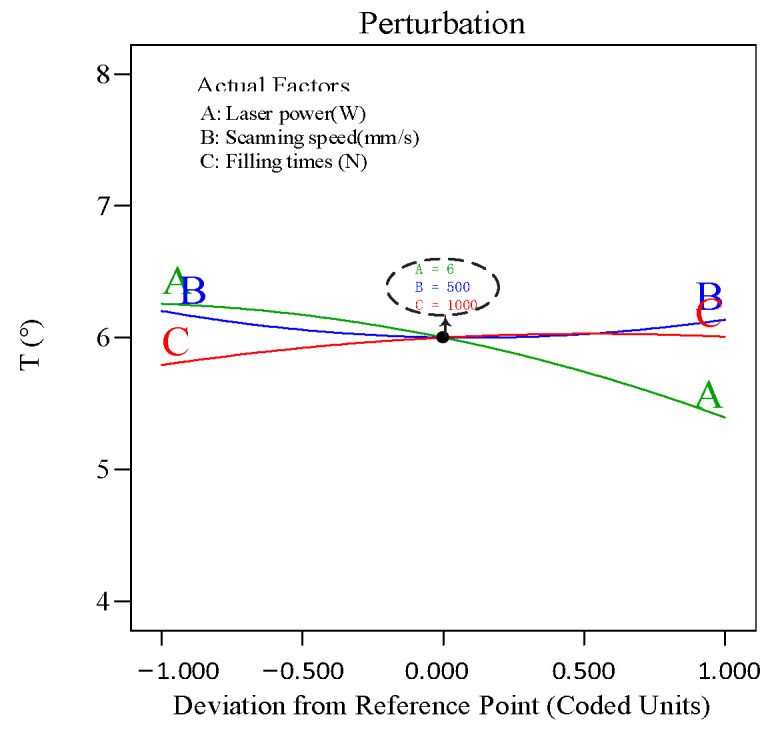
Perturbation plot for hole taper.

To analyze the effect of the process parameters and their interactions on hole taper, perturbation plots and response surface plots were used for evaluation. The perturbation plot shows the mapping relationship between individual process parameters and the response value. Figure 15 shows the interaction effects of laser power (A), scanning speed (B), and filling times (C) on hole taper. It can be seen from the figure that laser power (A) is the main factor affecting hole taper, with hole taper decreasing as power increases. As scanning speed (B) increases, the hole taper slightly decreases, but once the scanning speed reaches a certain threshold, the hole taper begins to increase. Under the influence of filling times (C), as the number of fills increases, the hole taper also increases.

**Figure 16:**
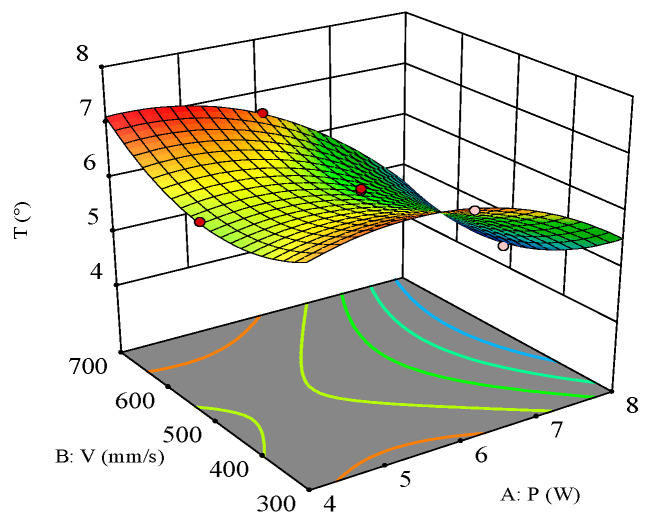
Three-dimensional surface plot for the effect of laser power and scanning speed on the hole taper.

As shown in Figure 16, the response surface of laser power and scanning speed on hole taper indicates that their interaction is significant. When the filling times are 1000 N, the influence patterns of laser power and scanning speed on the hole taper are observed. Higher average laser power and moderate scanning speed can achieve a smaller hole taper. Due to the characteristics of the laser, the energy density at the center is significantly higher than at the periphery, leading to faster material removal at the center of the spot compared to the surrounding material. When the power is low, the surrounding material receives less energy, making removal more difficult. Therefore, higher power removes more edge material, reducing the hole taper. Compared to low-power processing, higher power results in more ablation of the sidewalls, making it easier to remove sidewall material at deeper positions.

The influence pattern of scanning speed on the hole taper is relatively complex. The interaction of the laser with CFRP material produces both thermal and mechanical effects. Slower scanning speeds result in longer laser dwell times on the material surface, causing more material to evaporate and melt, thereby creating a larger heat-affected zone (HAZ). The expansion of the HAZ tends to increase the taper of the hole walls. As the scanning speed increases, the laser dwell time on the material surface shortens, reducing the HAZ and consequently decreasing the hole taper. However, as the scanning speed continues to increase, the energy received at each point further decreases, which may be insufficient to effectively remove material, resulting in decreased removal efficiency. At this point, the material removal capability is limited by the laser power and material properties, and the hole taper no longer changes significantly. Therefore, to achieve a smaller hole taper, the scanning speed should be moderate, and a higher laser power should be selected.

**Figure 17:**
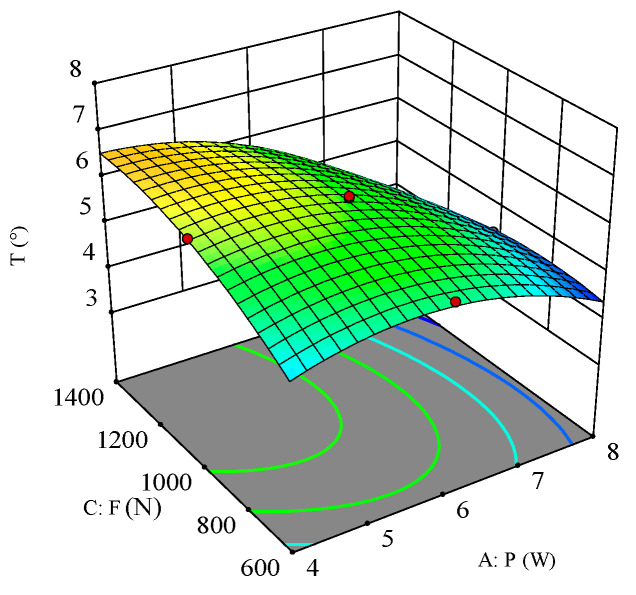
Three-dimensional surface plot for the effect of laser power and filling times on the hole taper.

When the scanning speed V = 500 mm/s, the effects of laser power and filling times on the hole taper are shown in Figure 17. The response surface plot indicates that the interaction between laser power and filling times is quite significant, and both have a negative correlation with the hole taper. It can be observed that higher average laser power and fewer filling times result in a smaller hole taper. As the number of filling times increases, the number of laser passes over the same area also increases, leading to local heat accumulation. This heat accumulation causes the material to locally melt and resolidify, which is particularly evident at the edge of the hole. This results in the upper wall material being more easily melted and removed, while the bottom material is less heated, forming a tapered hole. With the increase in filling times, the interaction mechanism between the laser and material may change from evaporation and ablation during the initial fillings to partial melting and resolidification of the material on the hole walls during subsequent fillings, especially in the lower region of the hole, leading to an increase in the hole taper.

**Figure 18:**
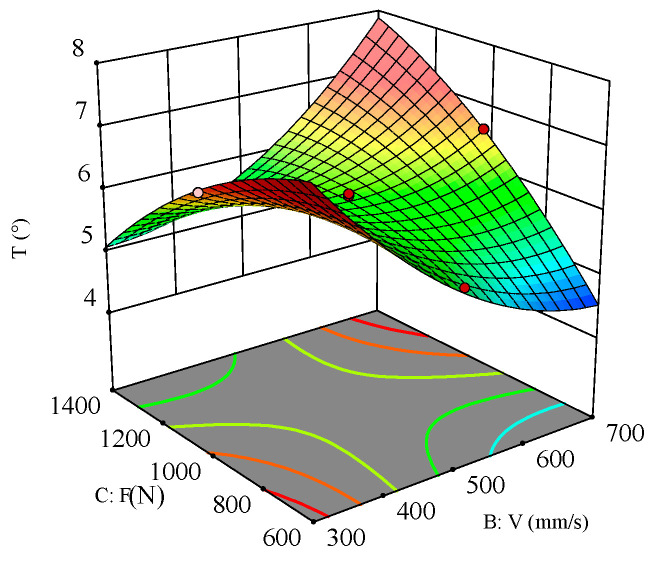
Three-dimensional surface plot for the effect of scanning speed and filling times on the hole taper.

Figure 18 shows the interaction effect of scanning speed and filling times on the hole taper. The response surface plot indicates that the interaction between scanning speed and filling times is quite significant, and both have a positive correlation with hole taper. When the laser power is fixed at 6 W, the hole taper decreases as the scanning speed and filling times increase.

#### 5.4.2. Effect of Process Parameters on the Heat-Affected Zone

**Figure 19:**
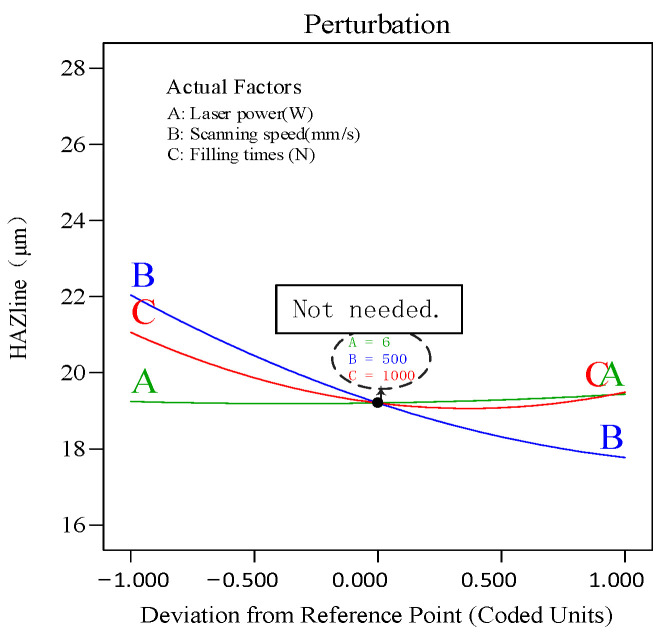
Perturbation plot for HAZ.

To analyze the effect of process parameters and their interactions on the hole taper, perturbation plots and response surface plots were used for evaluation. The perturbation plot shows the mapping relationship between individual process parameters and the response value. Figure 19 shows the interaction effects of laser power (A), scanning speed (B), and filling times (F) on the heat-affected zone (HAZ). It can be seen from the figure that scanning speed (B) is the main factor affecting the HAZ. As scanning speed increases, the HAZ decreases. As filling times (C) increase, the HAZ decreases, but once filling times exceed a certain threshold, the HAZ begins to increase. Under the influence of laser power (A), as it gradually increases, the HAZ also increases.

**Figure 20:**
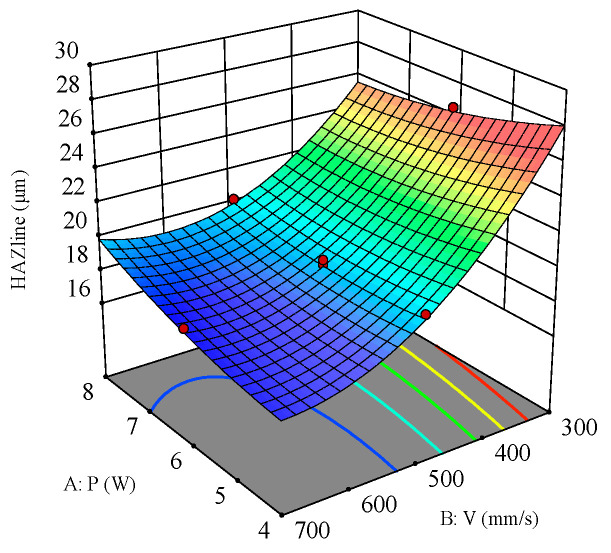
Three-dimensional surface plot for the effect of laser power and scanning speed on the HAZ.

As shown in Figure 20, the response surface of laser power and scanning speed on the heat-affected zone (HAZ) when the filling times are 1000 N indicates that their interaction is significant. It can be seen that with the filling times fixed, smaller laser power and higher scanning speed result in a smaller HAZ. The HAZ increases with increasing laser power due to the strong thermal and heat conduction effects caused by the increased energy density. Additionally, the high-power laser causes the material surface to melt and vaporize, and the resulting high-temperature region further affects the structure and properties of the surrounding material, enlarging the HAZ. The HAZ decreases with increasing scanning speed because the reduced dwell time of the laser on the material decreases local heat accumulation and heat diffusion effects. Furthermore, higher scanning speeds allow for more time for the material to cool naturally after the laser beam moves away, reducing heat accumulation and diffusion within the material and thus decreasing the HAZ.

**Figure 21:**
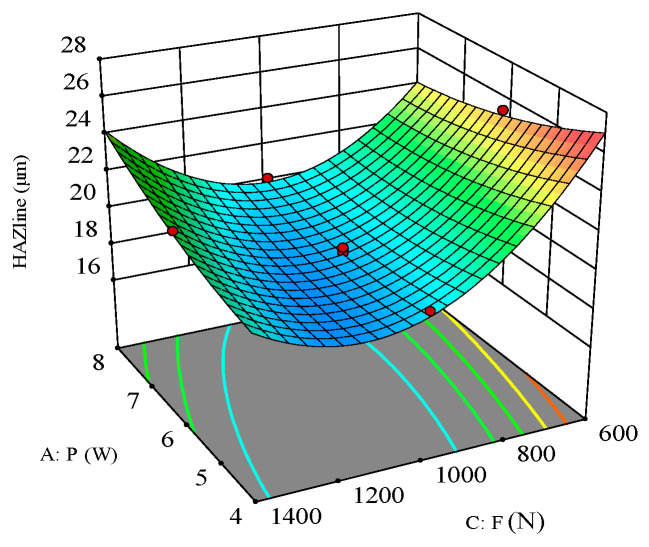
Three-dimensional surface plot for the effect of laser power and filling times on the HAZ.

When the scanning speed V = 500 mm/s, the effects of laser power and filling times on the heat-affected zone (HAZ) are shown in Figure 21. The response surface plot indicates that the interaction between laser power and filling times is quite significant, and both have a negative correlation with the HAZ. Increasing the number of filling times means that the same area receives more laser passes. However, since the filling method is concentric circles from outside to inside, the laser energy focuses more on the central region of the hole. Therefore, as the number of filling times increases, the HAZ does not increase as much as it did during the initial fillings. Instead, it decreases due to cooling and heat dispersion from multiple laser passes. Additionally, as the number of filling times increases, the thermal effect of the laser on the material becomes more evenly distributed across the entire processing area. This means that more of the material’s surface and volume are subjected to similar heat effects, reducing local temperature differences and further decreasing the size of the HAZ.

**Figure 22:**
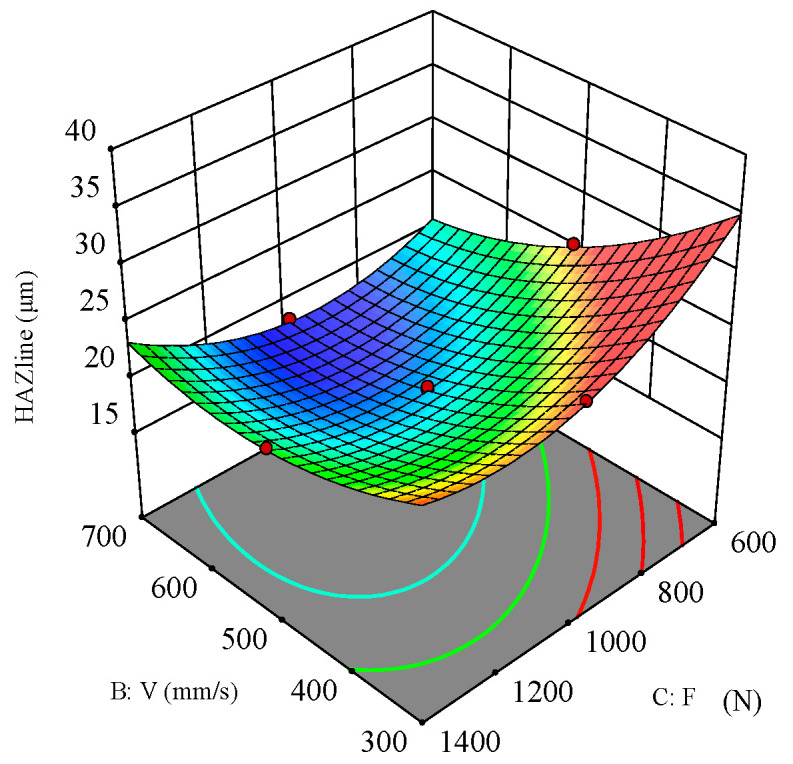
Three-dimensional surface plot for the effect of scanning speed and filling times on the HAZ.

Figure 22 shows the interaction effect of scanning speed and filling times on the heat-affected zone (HAZ). The response surface plot indicates that the interaction between scanning speed and filling times is quite significant, and both have a positive correlation with the HAZ. When the laser power is fixed, the HAZ decreases as scanning speed and filling times increase.

## 6. Conclusions

Based on the response surface method, a study was conducted on the drilling of carbon fiber-reinforced resin matrix composites using a 532 nm picosecond pulse laser. The effects of laser power, scanning speed, and filling times on processing quality were analyzed. The study conclusions are as follows:

(1) Four methods were studied for the quantitative characterization of hole taper and heat-affected zone size: hole taper (T_circle_, T_ellipse_) and heat-affected zone (HAZ_ellipse_, HAZ_line_). Through comparative analysis, it was found that the results of these four methods varied similarly with changes in processing parameters. To determine which method is more accurate and reliable, the process parameters were optimized using the response surface method, and these four sets of parameters were compared. Finally, Plan 3 (hole taper T_ellipse_, heat-affected zone HAZ_line_) was selected, with error rates of 5.0% and 2.2%, respectively.

(2) In the response surface analysis model, laser power has a significant impact on the hole taper. As laser power increases, hole taper gradually decreases. The factors affecting hole taper, in order of importance, are laser power > filling times > and scanning speed. For the heat-affected zone (HAZ), scanning speed is the most significant factor, followed by filling times and laser power. The HAZ decreases with increasing scanning speed and filling times.

(3) The optimized process parameters for laser drilling are a laser power of 7.886 W, a scanning speed of 633.514 mm/s, and filling times of 880.617 N. Experimental validation showed good macroscopic hole quality, with no cracks, pores, or other defects. The minimum hole taper achieved was 4.160° and the heat-affected zone was 18.577 μm.

(4) Due to resource and time constraints, our initial research focused on key process parameters. In the future, we plan to optimize the hole exit quality by expanding the analysis scope and technological innovation and comprehensively evaluating the impact of drilling parameters on the performance of composite materials to ensure the structural integrity of the components.

## Figures and Tables

**Table 1:** Typical thermal properties of matrix and fiber materials in CFRP structure.

Material Parameters	Carbon Fiber	Epoxy Resin
Volume Fraction	0.6	0.4
Density ρ (kg/m^3^)	1850	1200
Specific Heat Capacity C (J/(kg-K))	710	1884
Thermal Conductivity k (W/(m-K))	50 (axial), 5 (radial)	0.1
Latent Heat of Phase Change L (kJ/kg)	43,000	1000
Vaporization Temperature (K)	3900	698

**Table 2:** Characteristics of the experimental laser system and drilling parameters.

Characteristics	Symbols	Values	Units
Wavelength	λ	355	nm
Average laser power	*P_avg_*	≥30	w
Pulse energy	*E*	≥30	μJ
Pulse width	τ	≤7	ps
Laser power	p	4, 5, 6, 7, 8	W
Filling degree	T	600, 800, 1000, 1200, 1400	N
Scanning speed	V	300, 400, 500, 600, 700	mm/s

**Table 3:** Process parameters and factor levels.

Experiment Factor	Level				
	−2	−1	0	1	2
A: Laser power P (W)	4	5	6	7	8
B: Scanning speed V (mm/s)	300	400	500	600	700
C: Filling times F (N)	600	800	1000	1200	1400

**Table 4:** Experimental design parameters and results.

Run	Factors			Responses							
	Laser Power P/W	Scanning Speed V/(mm/s)	Filling Times F/(N)	Plan 1		Plan 2		Plan 3		Plan 4	
				Circle (°)	HAZ_line_ (μm)	Circle (°)	HAZ_ellipse_ (μm)	T_ellipse_(°)	HAZ_line_ (μm)	T_ellipse_ (°)	HAZ_ellipse_ (μm)
1	5	400	800	6.25751	25.2857	6.25751	35.5226	6.32119	25.2857	6.32119	35.5226
2	7	400	800	5.82421	23.741	5.82421	44.5422	6.00795	23.741	6.00795	44.5422
3	5	600	800	6.59224	18.5945	6.59224	22.22	5.71171	18.5945	5.71171	22.22
4	7	600	800	4.98998	18.9592	4.98998	28.7538	4.97568	18.9592	4.97568	28.7538
5	5	400	1200	6.11358	21.253	6.11358	40.8374	6.22256	21.253	6.22256	40.8374
6	7	400	1200	5.85383	21.8072	5.85383	38.9754	5.26934	21.8072	5.26934	38.9754
7	5	600	1200	7.00828	17.95	7.00828	37.2126	7.00553	17.95	7.00553	37.2126
8	7	600	1200	5.63907	19.447	5.63907	25.3778	5.7666	19.447	5.7666	25.3778
9	4	500	1000	6.2891	19.8093	6.2891	34.911	6.1975	19.8093	6.1975	34.911
10	8	500	1000	4.83957	20.1445	4.83957	36.3348	4.3681	20.1445	4.3681	36.3348
11	6	300	1000	6.79193	26.4928	6.79193	54.773	6.69944	26.4928	6.69944	54.773
12	6	700	1000	6.65339	17.9952	6.65339	32.382	5.61787	17.9952	6.61787	32.382
13	6	500	600	5.59202	25.3275	5.59202	26.6108	5.46267	25.3275	5.56267	26.6108
14	6	500	1400	5.66963	22.1163	5.66963	31.5374	5.69169	22.1163	5.69169	31.5374
15	6	500	1000	5.93751	19.337	5.93751	25.662	5.96703	19.337	5.96703	25.662
16	6	500	1000	5.71958	18.8185	5.71958	27	5.89491	18.8185	5.89491	27
17	6	500	1000	6.09909	19.47	6.09909	27.4224	6.08368	19.47	6.08368	27.4224
18	6	500	1000	5.99373	19.2524	5.99373	28.5636	5.9322	19.2524	5.7222	28.5636
19	6	500	1000	6.045	19.28	6.045	27.1296	6.118	19.28	5.918	27.1296
20	6	500	1000	5.875	19.547	5.875	27.7098	5.975	19.547	5.975	27.7098

**Table 5:** Optimization criteria and weights.

Technical Parameter		Criteria			Weight
		Goal	Lower Limit	Upper Limit	
A: Laser power P(W)		In range	4	8	1
B: Scanning speed V(mm/s)		In range	300	700	1
C: Filling times F(N)		In range	600	1400	1
Plan 1	T_circle_(°)	Minimize	4.83957	7.00828	1
	HAZ_line_(μm)	Minimize	17.95	26.4928	1
Plan 2	T_circle_(°)	Minimize	4.83957	7.00828	1
	HAZ_ellipse_(μm)	Minimize	22.22	54.773	1
Plan 3	T_ellipse_(°)	Minimize	4.3681	7.00553	1
	HAZ_line_(μm)	Minimize	17.950	26.4928	1
Plan 4	T_ellipse_(°)	Minimize	4.3681	7.00553	1
	HAZ_ellipse_(μm)	Minimize	22.22	54.773	1

**Table 6:** Optimization results.

Run	Parameter Combination			Predicted Value		Experimental Value		Error Rate	
	Laser Power P/W	Scanning Speed V/(mm/s)	Filling Times F/(N)	T_min_ (°)	HAZ_min_ (μm)	T_min_ (°)	HAZ_min_ (μm)	T_min_ (°)	HAZ_min_ (μm)
1	7.401	638.686	898.791	4.840	18.563	4.369	17.960	10.8%	3.4%
2	7.908	569.414	1399.999	4.815	24.245	4.436	37.774	8.6%	35.8%
3	7.886	633.514	880.617	4.368	18.982	4.160	18.577	5.0%	2.2%
4	4.000	668.937	600.000	4.463	22.216	11.961	21.445	62.68%	3.6%

**Table 7:** Variance analysis of the hole taper.

Source	Sum of Squares	df	Mean Square	F-Value	*p*-Value	
Model	6.64	9	0.7373	75.90	<0.0001	significant
A-P	2.98	1	2.98	306.31	<0.0001	
B-V	0.0172	1	0.0172	1.77	0.2128	
C-F	0.1818	1	0.1818	18.71	0.0015	
AB	0.0627	1	0.0627	6.46	0.0293	
AC	0.1633	1	0.1633	16.81	0.0021	
BC	1.07	1	1.07	109.85	<0.0001	
A^2^	0.7718	1	0.7718	79.45	<0.0001	
B^2^	0.7160	1	0.7160	73.70	<0.0001	
C^2^	0.2596	1	0.2596	26.72	0.0004	
Residual	0.0972	10	0.0097			
Lack of Fit	0.0590	5	0.0118	1.55	0.3218	not significant
Pure Error	0.0381	5	0.0076			
Cor Total	6.73	19				
	R^2^ = 0.9856			Adjusted R^2^ = 0.9726		
	Predicted R^2^ = 0.9193			Adeq Precision = 36.6883		

**Table 8:** Variance analysis of the heat-affected zone.

Source	Sum of Squares	df	Mean Square	F-Value	*p*-Value	
Model	124.88	9	13.88	111.35	<0.0001	significant
A-P	0.1485	1	0.1485	1.19	0.3006	
B-V	72.81	1	72.81	584.26	<0.0001	
C-F	9.84	1	9.84	78.94	<0.0001	
AB	1.02	1	1.02	8.16	0.0171	
AC	1.31	1	1.31	10.47	0.0089	
BC	4.22	1	4.22	33.86	0.0002	
A^2^	0.4539	1	0.4539	3.64	0.0854	
B^2^	12.36	1	12.36	99.18	<0.0001	
C^2^	28.82	1	28.82	231.26	<0.0001	
Residual	1.25	10	0.1246			
Lack of Fit	0.9219	5	0.1844	2.84	0.1381	not significant
Pure Error	0.3243	5	0.0649			
Cor Total	126.13	19				
	R^2^ = 0.9901			Adjusted R^2^ = 0.9812		
	Predicted R^2^ = 0.9397			Adeq Precision = 34.1836		

## Data Availability

The original contributions presented in the study are included in the article, further inquiries can be directed to the corresponding author.
